# Clinical outcomes and effect on intraoperative blood loss and postoperative pain of patients undergoing retroperitoneal laparoscopic partial nephrectomy for complex renal tumors

**DOI:** 10.1186/s12957-021-02397-x

**Published:** 2021-09-18

**Authors:** Yansong Guo, Qian Xu, Baochun Chen, Lifeng Liu, Yuanyuan Wang, Ai Zhu, Longjiang Tian

**Affiliations:** 1 Department of Urology Surgery, Cangzhou People’s Hospital, 1401 unit 1, building 5, Huayuan Yishijie, Yunhe District, Cangzhou City, Hebei Province China; 2 Department of Anesthesiology, Cangzhou People’s Hospital, Cangzhou, 061000 Hebei Province China; 3 Department of Internal Medicine-Neurology, Cangzhou Central Hospital, Cangzhou, 061000 Hebei Province China

**Keywords:** Retroperitoneal laparoscopic partial nephrectomy, Complex renal tumor, Clinical outcome, Intraoperative blood loss, Postoperative pain

## Abstract

**Objective:**

To explore the clinical outcomes and effect on intraoperative blood loss and postoperative pain of patients undergoing the retroperitoneal laparoscopic partial nephrectomy (RLPN) for complex renal tumors.

**Methods:**

Fifty patients with complex renal tumor admitted to our hospital from February 2017 to February 2019 were selected as the research object and divided into the RLPN group (given the retroperitoneal laparoscopic partial nephrectomy, *n* = 24) and the OPN group (given the open partial nephrectomy, *n* = 26) by number table method to compare their various perioperative indicators and serum stress response and analyze the clinical effect of different surgical methods on the complex renal tumor.

**Results:**

The clinical information of patients in both groups were not significantly different (*P* > 0.05); in addition to the operative time, the intraoperative blood loss, hospital stay, warm ischemia time, and numerical rating scale (NRS) scores of the RLPN group were clearly lower than those of the OPN group (*P* < 0.05); after treatment, patients in the RLPN group obtained significantly lower white blood cell (WBC) count, cortisol, and c-reactive protein (CRP) levels than the OPN group (*P* < 0.05); the renal glomerular filtration rate (GFR) of the affected side, quality of life scores, and 3-year overall survival rate of treated patients in the RLPN group were obviously higher than those in the OPN group (*P* < 0.05); and patients in the RPLN group had significantly lower incidence rate (*P* < 0.05).

**Conclusion:**

Compared with OPN, RLPN is more worthy of promotion and application, because it has better treatment outcomes, significantly reduces intraoperative blood loss, alleviates the body stress response and postoperative pain, and improves the quality of life.

## Background

The renal tumor is a common type of urinary system tumors with the incidence rate that ranks only second to bladder cancer. As the biological behavior capacity of renal tumor is low, such disease is relatively less malignant and less prone to metastasis, with well-defined lesion borders and slow growth [1, 2]. The continuous progress of medical diagnosis techniques in recent years has improved the early diagnosis rate of renal tumors and realized a desirable clinical prognosis. Clinical studies have confirmed [3] that renal tumors are resistant to a variety of drugs and less sensitive to radiation, leading to limited biological targeting and immunotherapy, so surgical intervention becomes the most effective treatment modality for renal tumors. Nephron-sparing surgery (NSS), a type of surgery currently advocated in clinical treatment of renal tumors [4, 5], not only effectively preserves patients’ nephrons but also prolongs patients’ survival time to some extent. Complex renal tumors refer to localized tumors with RENAL score ≥ 7, a functional solitary or anatomic kidney, and no clinical and biological features such as local or distant metastasis [6]. Therefore, it is difficult to enucleate or excise complex renal tumors while preserving the nephron because of the deep encapsulation of renal parenchyma and the relatively close proximity to the anatomically complex renal collecting system. As research has progressed, surgical methods including retroperitoneal laparoscopic partial nephrectomy (RLPN), robot-assisted partial nephrectomy (RAPN), and open partial nephrectomy (OPN) have been widely applied in various renal tumor diseases, thus expanding the scope of indications [7–9]. In this study, we compared the clinical efficacy of RLPN with OPN in the treatment of complex renal tumors and analyzed the effect on intraoperative blood loss and postoperative pain in patients, as reported as follows.

## Materials and methods

### General information

Totally, 50 patients with complex renal tumor admitted to our hospital from February 2017 to February 2019 were selected as the research object and divided into the RLPN group (*n* = 24) and the OPN group (*n* = 26) by number table method. The study was approved by the Hospital Ethics Committee, and all patients signed the informed consent.

### Inclusion criteria

The inclusion criteria are as follows: (1) the patients were diagnosed as complex renal tumor by intravenous pyelogram and other imaging examinations; (2) no chemotherapy or other tumor related treatment was given before operation; (3) the renal function indexes such as serum creatinine and urea nitrogen were normal before operation; and (4) the study was approved by the Hospital Ethics Committee, and the patients have signed the informed consent.

### Exclusion criteria for the patients

The exclusion criteria are as follows: (1) those who presented with lymph node distant metastasis by the imaging examination, (2) those with a previous history of renal surgery, and (3) those who received other renal interventions or died or lost during follow-up.

### Methods

After admission, related physical examination was conducted to patients in both groups, and clinical health education and psychological intervention were carried out, including advising the patients to accept the surgery with a good mood and informing their family members of daily precautions. Both surgeries were performed by the same group of doctors. The specific steps of performing the OPN treatment were as follows: patients were lying on the healthy side to perform general anesthesia with endotracheal intubation and then had their waist raised after routine disinfection and draping, a 12–15-cm-long incision was made at the lower edge of the 12th rib or between the 11th rib via retroperitoneal approach, the tissues were incised layer by layer to fully expose the peri-renal fascia for routine probing by a self-retractor, and then the peritoneum was pushed forward appropriately to cut open the gerota fascia and separate the peri-renal fascia with the fat layer by layer; after that, the renal artery and the kidney were freed to fully expose the tumor lesion, the renal capsule was cut open by an electrocoagulation knife at 5 mm away from the lesion edge and the excision extension was marked, the renal artery was blocked by the Bulldog vascular clamps, and then the tumor lesion was completely excised along the marking line, as well as partial normal renal parenchymal tissue; after that, the wound was sutured and covered with gauze to stop bleeding, the Bulldog clamps were taken out to observe for signs of bleeding, a drainage tube was indwell, the incision was closed up, and the wound was bound up [7–9].

The specific steps of performing the RLPN treatment were as follows: patients were lying on the healthy side to perform general anesthesia and had their waist bridge raised to perform routine disinfection and draping and then establish the operation space at retroperitoneum; the operation channels were set up at 2 transverse fingers from anterior superior iliac spine crest at the midaxillary line, under the costal margin of anterior axillary line, and under the costal margin of posterior axillary line while the pneumoperitoneum pressure was maintained at about 12 mmHg; the intraperitoneal environment was inspected, and the extraperitoneal adipose tissue was removed with the ultrasonic knife; the peri-renal fascia was cut open and separated to expose the dorsal kidney, separate the renal pedicle, free the kidney artery and the kidney, and fully expose the tumor lesion, and then, the excision, stopping the bleeding, suture, and indwelling of drainage tube were performed according to the same steps as OPN. The patients in both groups received the anti-infection and nutritional support treatment after operation. When the postoperative drainage volume was less than 10 ml for 3 consecutive days, the drainage tube was removed. And outpatient or telephone follow-up was given to patients in both groups for 3 years [10–12].

### Observation indexes

Various perioperative clinical indicators, including the operative time, intraoperative blood loss, hospital stay, and warm ischemia time, were recorded and compared, and the pain at 48 h after surgery of patients in both groups was evaluated by the 0–10 numerical rating scale (NRS) [13] for pain, with higher scores indicating more serious pain.

The serum stress response indexes such as the white blood cell (WBC) count, c-reactive protein (CRP), and cortisol levels of patients in both groups were measured before and after treatment.

The renal glomerular filtration rate (GFR) was measured by the multi-phase enhanced spiral CT scan before and after treatment;

The quality of life of patients in both groups was evaluated by the *European Organization for Research and Treatment of Cancer Quality of Life Questionnaire* (EORTC QLQ) [14] before and after treatment, and the maximum score was 100 points, with higher scores indicating higher quality of life.

The clinical adverse reactions and 3-year survival rate of patients in both groups were counted and compared after surgery.

### Statistical methods

The statistical analysis was conducted with SPSS21.0, the picture drawing of data was completed by GraphPad Prism 7 (GraphPad Software, San Diego, USA), the enumeration data were examined by *χ*^2^ test and expressed by [*n*(%)], the measurement data were examined by *t* test and expressed by ($\overline{\mathrm{x}}$±s), and differences were considered statistically significant at *P* < 0.05.

## Results

### Comparison of patients’ clinical information between the two groups

The sex ratio, mean age, mean BMI value, tumor location, tumor staging, tumor diameter, and place of residence of patients in both groups were not statistically different (*P* > 0.05) but comparable; see Table 1.

**Table 1:** Comparison of patients’ clinical information between the two groups

Category	RLPN group (*n* = 24)	OPN group (*n* = 26)	*χ*^2^/*t*	*P*
Gender			0.002	0.963
Male	14 (58.33%)	15 (57.69%)		
Female	10 (41.67%)	11 (42.31%)		
Mean age (years old)	52.31 ± 4.61	53.18 ± 4.52	0.674	0.504
Mean BMI (kg/m^2^)	21.15 ± 1.03	21.23 ± 0.96	0.284	0.777
Tumor location			0.001	0.982
Left side	13 (54.17%)	14 (53.85%)		
Right side	11 (45.83%)	12 (46.15%)		
Tumor staging			0.244	0.621
*T*_1a_	16 (66.67%)	19 (73.08%)		
*T*_1b_	8 (33.33%)	7 (26.92%)		
Tumor diameter (cm)	3.16 ± 0.53	3.23 ± 0.48	0.490	0.626
Place of residence			0.002	0.963
Urban area	10 (41.67%)	11 (42.31%)		
Rural area	14 (58.33%)	15 (57.69%)		

### Comparison of patients’ perioperative clinical indicators between the two groups

In addition to the operative time, the intraoperative blood loss, hospital stay, warm ischemia time, and NRS scores of the RLPN group were significantly lower than those of the OPN group (*P* < 0.05); see Table 2.

**Table 2:** Comparison of patients’ perioperative clinical indicators between the two groups ($\overline{\mathrm{x}}$ ± s)

Group	*n*	Operative time (min)	Intraoperative blood loss (mL)	Hospital stay (days)	Warm ischemia time (min)	NRS score (points)
RLPN group	24	102.35 ± 8.92	100.25 ± 10.72	8.24 ± 1.47	18.94 ± 4.52	3.17 ± 0.47
OPN group	26	95.61 ± 9.23	126.71 ± 9.84	10.18 ± 1.34	23.41 ± 4.61	5.09 ± 0.61
*t*		2.621	9.101	4.882	3.458	12.391
*P*		< 0.05	< 0.001	< 0.001	< 0.05	< 0.001

### Comparison of serum stress response indicators before and after treatment between the two groups

After treatment, the WBC, cortisol, and CRP levels of patients in the RLPN groups were significantly lower than those in the OPN group (*P* < 0.05); see Table 3.

**Table 3:** Comparison of serum stress response indicators before and after treatment between the two groups ($\overline{\mathrm{x}}$ ± s)

Group	*n*	WBC (× 10^9^)		Cortisol (μmol/L)		CRP (mg/L)	
		Before	After	Before	After	Before	After
RLPN group	24	5.53 ± 1.24	6.17 ± 1.18	0.47 ± 0.14	0.63 ± 0.14	7.62 ± 0.19	9.85 ± 2.14
OPN group	26	5.57 ± 1.29	7.24 ± 1.12	0.51 ± 0.16	0.89 ± 0.23	7.67 ± 0.15	12.37 ± 2.08
*t*		0.112	3.289	0.937	4.779	1.037	4.221
*P*		0.912	< 0.05	0.353	< 0.001	0.305	< 0.001

### Comparison of renal GFR levels of the affected side before and after treatment between the two groups

**Fig. 1:**
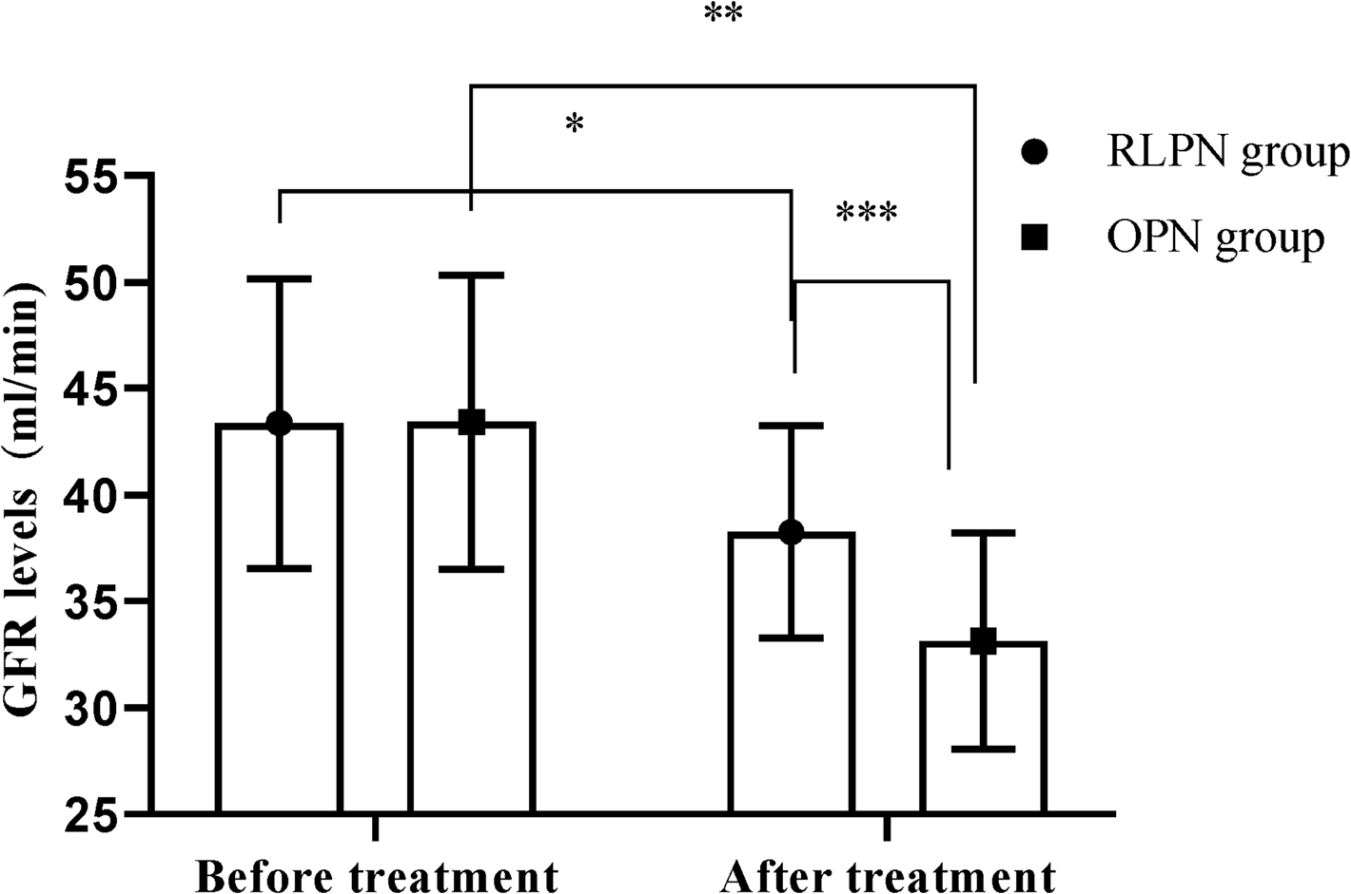
Comparison of renal GFR levels of the affected side before and after treatment between the two groups ($\overline{\mathrm{x}}$ ± s). The horizontal axis indicated before and after treatment, and the vertical axis indicated the GFR level values in ml/min; before and after treatment, the patients’ renal GFR level values of the affected side of the RLPN group were 43.37 ± 6.83 and 38.26 ± 4.97, respectively, and those of the OPN group were 43.42 ± 6.91 and 33.14 ± 5.07, respectively; the single asterisk (*) symbol indicated that before and after treatment, the renal GFR level values of the affected side of the RLPN group were clearly different (*t* = 2.964, *P* < 0.05); double asterisk (**) symbol indicated that before and after treatment, the renal GFR level values of the affected side of the OPN group were clearly different (*t* = 6.116, *P* < 0.001); and triple asterisk (***) symbol indicated that after treatment, the renal GFR level values of the affected side of both groups were clearly different (*t* = 3.601, *P* < 0.05)

After treatment, the renal GFR levels of the affected side of both groups were obviously lower than those before treatment, and in the between-group comparison, the RLPN group was significantly higher than the OPN group (*P* < 0.05); see Fig. 1.

### Comparison of quality of life scores before and after treatment between the two groups

**Fig. 2:**
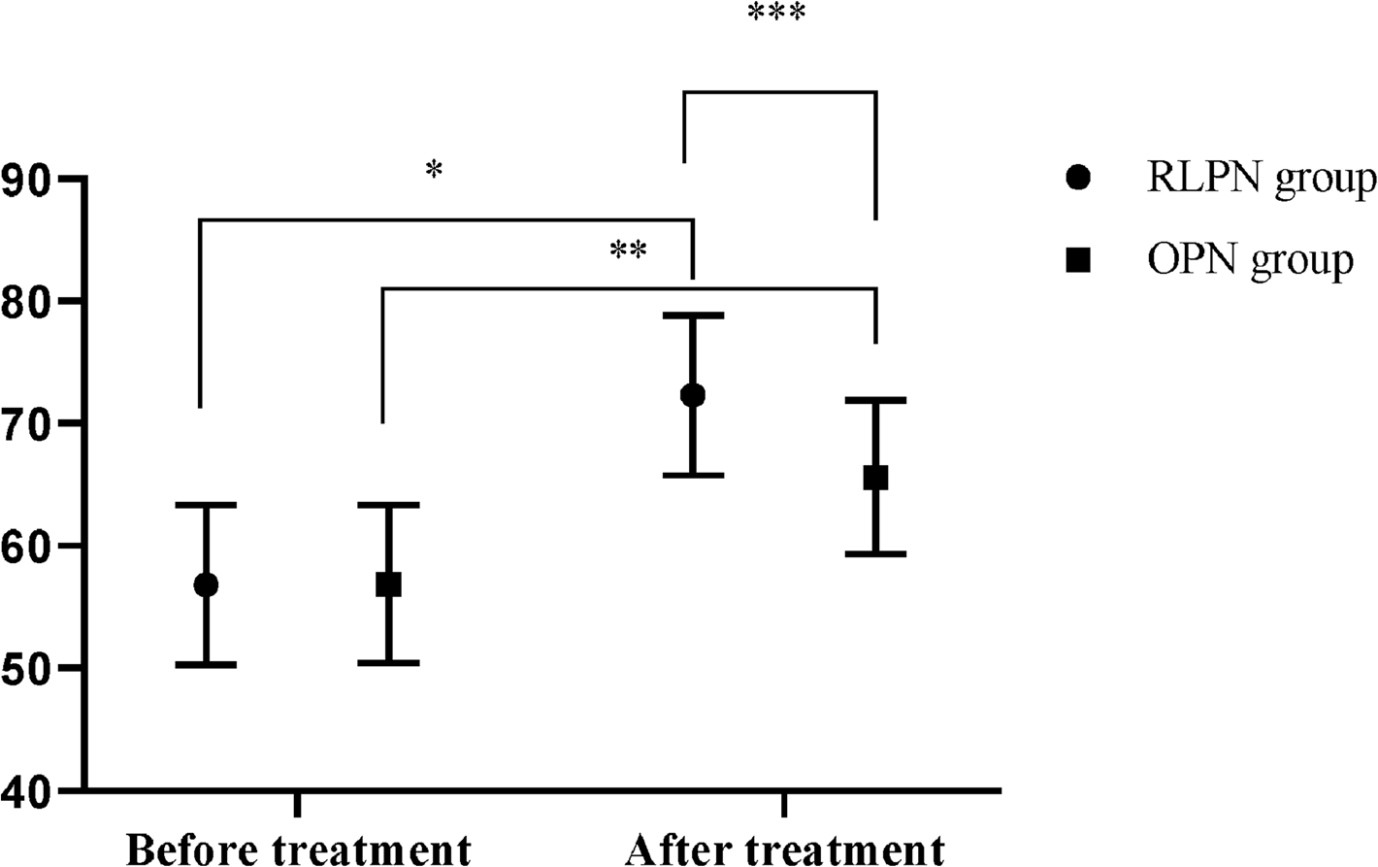
Comparison of quality of life scores before and after treatment between the two groups ($\overline{\mathrm{x}}$ ± s). The horizontal axis indicated before and after treatment, and the vertical axis indicated the quality of life scores (points); before and after treatment, the patients’ quality of life scores of the RLPN group were 56.81 ± 6.54 and 72.31 ± 6.52, respectively, and those of the OPN group were 56.87 ± 6.48 and 65.61 ± 6.27, respectively; the single asterisk (*) indicated that before and after treatment, the patients’ quality of life scores of the RLPN group were clearly different (*t* = 8.223, *P* < 0.001); double asterisk (**) symbol indicated that before and after treatment, the patients’ quality of life scores of the OPN group were clearly different (*t* = 4.942, *P* < 0.001); and triple asterisk (***) symbol indicated that after treatment, the patients’ quality of life scores of both groups were clearly different (*t* = 3.704, *P* < 0.05)

After treatment, the quality of life scores of both groups were significantly higher than those before treatment (*P* < 0.05), and in the between-group comparison, the RLPN group was significantly higher than the OPN group (*P* < 0.05); see Fig. 2.

### Comparison of postoperative complications incidence between the two groups

After operation, the incidence rate of postoperative complications of the RLPN group was significantly lower than that of the OPN group (*P* < 0.05); see Table 4.

**Table 4:** Comparison of postoperative complications incidence between the two groups [*n*(%)]

Group	*n*	Urinary fistula	Delayed hemorrhage	Urinary system infection	Hematuresis	Total incidence rate
RLPN group	24	0 (0.00)	1 (4.17)	0 (0.00)	0 (0.00)	4.17% (1/24)
OPN group	26	2 (7.69)	1 (3.85)	2 (7.69)	2 (7.69)	26.92% (7/26)
*χ*^2^						4.809
*P*						0.028

### Comparison of patients’ survival curves between the two groups

**Fig. 3:**
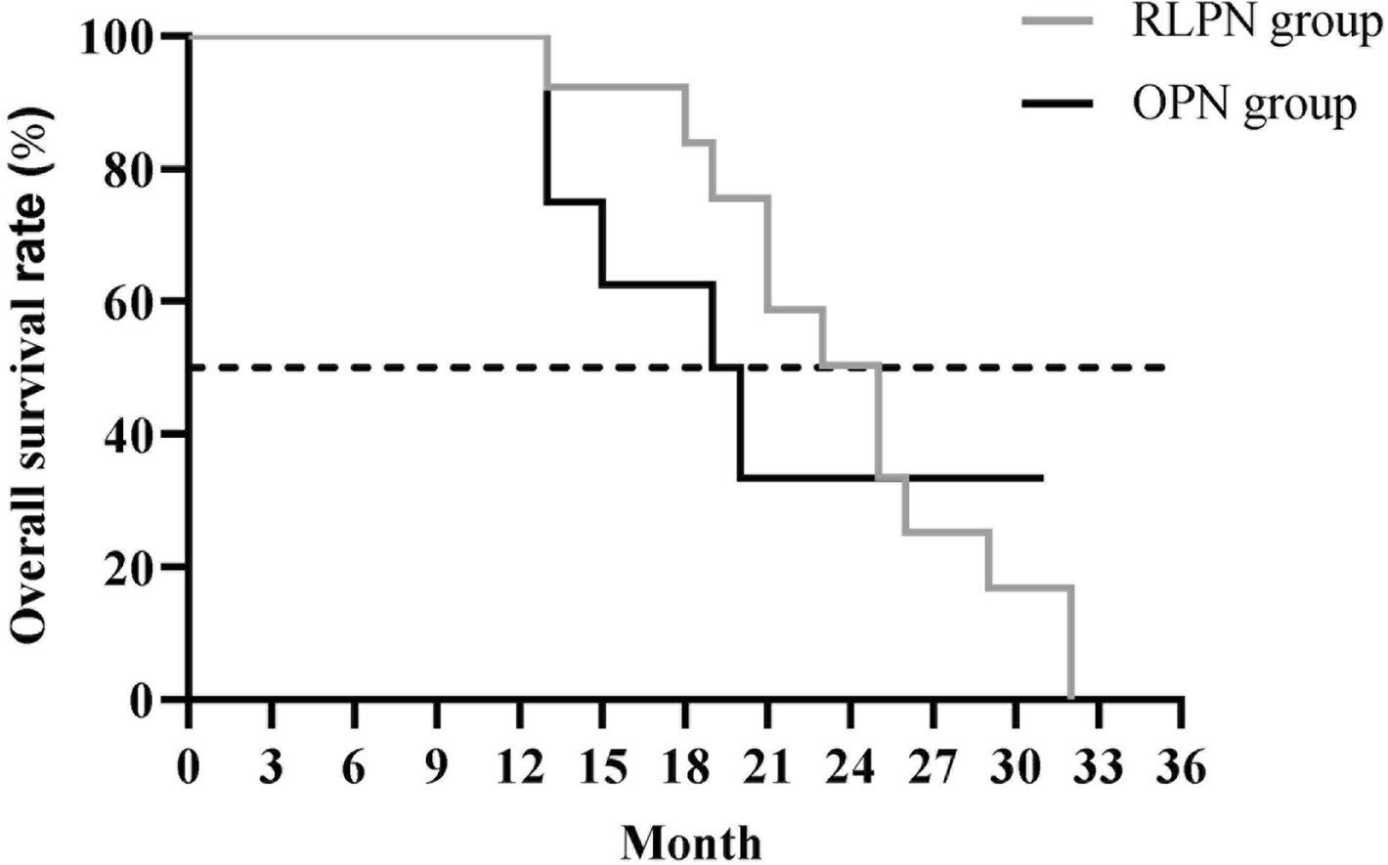
Comparison of patients’ survival curves between the two groups. The horizontal axis indicated the survival time in month, the vertical axis indicated the survival rate, and the survival curves of the OPN group and RLPN group were indicated by the black line and the light gray line, respectively

The study showed that in the RLPN group, the median survival time was 25 months and 21 cases survived with the survival rate of 87.50% (21/24), and in the OPN group, the median survival time was 20 months and 16 cases survived with the survival rate of 61.54% (16/26). The 3-year overall survival rate of the RLPN group was significantly higher than that of the OPN group by the Log-rank method (*P* < 0.05); see Fig. 3.

## Discussion

Difficulty in tumor resection and clinical efficacy are closely related to the anatomical situation of tumors, especially for patients with renal tumors, and the tumor location and size directly determine the proportion of nephron sparing when designing the NSS procedure and the complexity of operation [15, 16]. In China, the R.E.N.A.L nephrometry scoring system is usually adopted for renal tumor assessment, and the scores not less than 7 points are defined as complex renal tumors. Although this scoring system plays a part for proposing surgical ideas and predicting postoperative renal function of patients, it never presents the exact and uniform criteria for surgical selection in the clinic so far. Therefore, the surgical treatment of complex renal tumors should be further confirmed [17, 18]. With the continuous application of laparoscopic technology in recent years, laparoscopic surgical resection has become the first choice for the clinical treatment of renal cancer. A large number of literature have reported that RLPN has a better resection outcome than the open surgery in treating renal tumors not less than 4 cm in diameter [19, 20], because laparoscopy can provide doctors with a clear view of operation for comprehensively observing and magnifying the lesions, so that the intraoperative tissue dissection and lesion resection are more accurate and the surgical operation damage to surrounding tissues and nerves is reduced. At the same time, using the ultrasonic knife can quickly excise the lesions, reduce intraoperative blood loss, and more benefit the recovery of intestinal function in patients, thus shortening the hospital stay [21, 22]. However, despite all the advantages of RLPN, it still has the disadvantages of longer learning curve, higher demand for doctors’ operational level, and longer operative time.

The results of the study showed that except for the operative time, the RLPN group obtained significantly lower intraoperative blood loss, hospital time, warm ischemia time, and NRS scores than the OPN group (*P* < 0.05), indicating that although the operative time of RLPN was longer because of the difficulty, the patients’ intraoperative blood loss and postoperative pain were greatly reduced, which was good for recovery. In addition, the patients in the OPN group had significantly higher serum stress response indicators than the RLPN group after treatment (*P* < 0.05), because the incision made between the 11th or the 12th rib to enter the kidney and realize partial nephrectomy to patients in the OPN group was more traumatic, thus leading to strong stress on the body and easily triggering serious complications that affect the postoperative rehabilitation [23]. Fuat Kızılay and others [24] pointed out in their study that “by comparing the clinical effect of retroperitoneal laparoscopic partial nephrectomy with open partial nephrectomy on the treatment of localized renal cancer, it was found that the postoperative CRP levels of patients undergoing RLPN and OPN were (9.14 ± 2.53) mg/L and (12.76 ± 2.47) mg/L, which presented a significant difference,” demonstrating that the open surgery would increase the stress response on the body and influence postoperative recovery

## Conclusions

To sum up, compared with OPN, RLPN is more worthy of promotion and application, because it has better treatment outcomes, prolongs the survival time of patients, alleviates the stress response on the body, and improves the quality of life.

## Data Availability

The datasets used and/or analyzed during the current study are available from the corresponding author on reasonable request.
